# Turning glucosinolate into allelopathic fate: investigating allyl isothiocyanate variability and nitrile formation in eco-friendly *Brassica juncea* from South Korea

**DOI:** 10.1038/s41598-024-65938-w

**Published:** 2024-07-04

**Authors:** Da-Yeong Ko, Su-Mi Seo, Yong-Hyuk Lee, Chan Saem Gil, Hojoung Lee, Kang-Mo Ku

**Affiliations:** 1 Department of Plant Biotechnology, College of Life Sciences and Biotechnology, Korea University, 145 Anam-Ro, Seongbuk-Gu, Seoul, 02841 Republic of Korea; 2 Department of Horticulture, Chonnam National University, Gwangju, Republic of Korea 61186; 3 Agricultural Technology Center of Yeosu City, Yeosu, 59633 Republic of Korea; 4 Department of Horticulture, College of Industrial Science, Kongju National University, Yesan, 32439 Republic of Korea

**Keywords:** Glucosinolate, Sinigrin, Allyl isothiocyanate, Mustard, Epithiospecifier protein, Biochemistry, Plant sciences, Ecology

## Abstract

Leaf mustard (*Brassica juncea* L.) is explored for its biofumigant properties, derived from its secondary metabolites, particularly allyl isothiocyanate (AITC), produced during the enzymatic breakdown of glucosinolates like sinigrin. The research examines eight leaf mustard cultivars developed in Yeosu city, South Korea, focusing on their genetic characteristics, AITC concentration and nitriles formation rates from glucosinolates. Results indicate that the allelopathic effects, largely dependent on AITC concentration and enzymatic activity, vary across cultivar. Sinigrin and AITC constitute 79% and 36%, respectively, of glucosinolate and its hydrolysis products. The cultivar 'Nuttongii' demonstrates significant potential for inhibiting weeds, exhibiting the highest AITC concentration at 27.47 ± 6.46 µmole g^−1^ These outcomes highlight the importance of selecting mustard cultivars for biofumigation based on their glucosinolate profiles and hydrolysis product yields. The study also identifies a significant genetic influence on AITC and nitrile formation, suggesting that epithiospecifier protein modulation could enhance both allelopathic and other beneficial effects. Collectively, the research underscores the promise of mustard as a sustainable, environmentally friendly alternative to traditional herbicides.

## Introduction

Biofumigation refers to the biochemical processes within plants that result in the production of secondary metabolites, thereby contributing to their allelopathic effects on surrounding organisms^1,2^. As the challenge of herbicide-resistant weeds continues to expend, biofumigation stands out as an eco-friendly alternative in agriculture, harnessing the power of cover crops^3,4^. Biofumigation not only addresses the issue of herbicide resistance but also brings about notable impacts, including changes in soil pH attributed to the persistent use of herbicide^5,6^.

The Brassica family, particularly Brown mustard (*Brassica juncea*), is well known for its production of chemicals known as isothiocyanates by previous studies^7^. The brown mustard, commonly known as leaf mustard, has been utilized for pest and bacteria suppression^8^. The unique ability of leaf mustard to generate isothiocyanates underscores its significance not only in culinary, where it contributes to the distinctive taste and spiciness of the plant, but also as a valuable resource for pest and bacterial control in various agricultural systems. Nevertheless, further research is required to effectively implement this concept in weed control, particularly in understanding the interactions between allelochemicals and weeds. Leaf mustard, for instance, is rich in glucosinolates, especially sinigrin, which is recognized as the origin of its unique taste and spiciness^9^. Physical damage to the tissues of leaf mustard serves as a catalyst, initiating an enzymatic reaction involving myrosinase. This enzyme is responsible for the conversion of sinigrin into allyl isothiocyanate (AITC)^10,11^. AITC, a compound found in leaf mustard, has been observed to exhibit a range of biological effects, including anti-cancer, anti-obesity, antiviral, and antibacterial properties. These effects arise primarily due to its capacity to inhibit the cell growth cycle^12–14^. Consequently, the cell cycle arrest attribute of leaf mustard enhances its value as a cover crop^15^. Previous studies have shown that AITC can cause damage to the root hairs of lettuce seeds, leading to drought stress and significantly inhibiting plant growth at higher concentrations^16^. The presence and concentration of epithiospecifier protein (ESP) in plant tissues are pivotal in determining whether the hydrolysis of glucosinolates results in the production of AITC or epithionitriles, thus affecting the profile of allelopathic compounds produced^17^. Despite various studies on mustard, the knowledge about the enzymatic reactions and hydrolysis products in relation to ESP is still not fully understood. Moreover, although there have been studies, such as those by Frazie, et al.^18^, on glucosinolate and its hydrolysis products in leaf mustard, research specifically focusing on their allelopathic effects is comparatively limited. Hence, the goal of this study is to clarify the allelopathic effects and potential applications of various mustard cultivars. The Ministry of Agriculture, Food, and Rural Affairs of South Korea has been developed a genetic resource collection project aimed at developing new leaf mustard cultivars, including ‘Nuttongii’, ‘Sundongii’, ‘Sindongii’, ‘Zzangdolii’, ‘Ssamdolii’, ‘Kkottolii’, ‘Alsami’, and ‘Mekomi’ since 2001. The allelopathic potential of leaf mustard varieties bred in Yeosu city has not been extensively studied. There is a necessity for comprehensive laboratory-scale research to establish the suitable concentration for utilizing these cultivars as cover crops. Previous research has revealed the biofumigation effects of leaf mustard, but there remains a scarcity of information regarding the allelopathic potential of diverse leaf mustard cultivars^19^. This study delves into the genetic diversity of mustard compounds with a focus on their allelopathic characteristics. This inquiry is crucial in identifying the most suitable mustard cultivar for use as a cover crop. It also involves a comparison between the widely utilized ‘Dolsan’ mustard variety, which is the most popular cultivar, and eight locally bred cultivars from Yeosu City. The objective is to underscore the potential of Korean mustard varieties as biofumigation agents, presenting an innovative approach to weed management. This comparative analysis is vital for understanding the variations in effectiveness and possible applications in agricultural methodologies.

## Materials and methods

### Plant cultivation and harvest

Eight leaf mustard cultivars (‘Nuttongii’, ‘Sundongii’, ‘Sindongii’, ‘Zzangdolii’, ‘Ssamdolii’, ‘Kkottolii’, ‘Alsami’, and ‘Mekomi’) and the common ‘Dolsan’ cultivar mustard (*Brassica juncea* Coss, Worldseed Agricultural Corporation, Gyeonggi-do, Republic of Korea), were sown into 50-cell seedling trays and incubated in green house at average of 24 °C. After two weeks, at 3 March 2021, the seedlings were transplanted in a randomized block design with three replications per cultivar. The cultivars were conventionally cultivated at the Chonnam National University farm in Naju, Jeollanam Province, Republic of Korea (34°58′28.4″N, 126°45′59.3″E) until the time of harvest. Leaf mustards were harvested at the bolting stage in accordance with prior research findings, which have indicated that the highest sinigrin concentration is typically observed at this bolting stage^20^. The harvest dates for leaf mustard cultivars were categorized based on their bolting times, with harvests occurring on April 30, 2021, or May 21, 2021. Given that the average cultivation period for leaf mustard is 60 days, the cultivars were classified according to whether bolting occurred within the 60-day cultivation period from the transplanting date or after this period. Cultivars that bolted within 60 days from transplanting to harvest were classified as the early-harvested group (‘Dolsan (control)’, ‘Alsami’, ‘Sundongii’, ‘Kkottolii’), while those that bolted after 60 days were classified as the late-harvested group (‘Nuttongii’, ‘Mekomi’, ‘Sindongii’, ‘Zzangdolii’ ‘Ssamdolii’). Morphological traits including days to flowering, bolting rate, leaf length, number of leaves, fresh weight, and days to maturity were determined at day of harvest.

### Plant material preparation

On the day of harvest, six undamaged plants were selected from each replication. The longest leaf length, number of leaves, and the fresh weight were measured. For sample collection, a total of four fresh mustard plants per each replication (*n* = 3) were removed the dirt, chopped, and pooled together. The samples were immediately frozen using liquid nitrogen and then freeze-dried at − 80 °C under 5 mTorr pressure (MCFD8508, IlshineBioBase Co. Ltd., Dongducheon, Korea). The freeze-dried leaf mustard samples were ground using a coffee grinder (BCG-620SP, Bean Cruise, Seoul, Korea) and stored in a freezer at − 20 °C until further analysis.

### Allelopathic bioassay in agar petri dish

**Figure 1:**
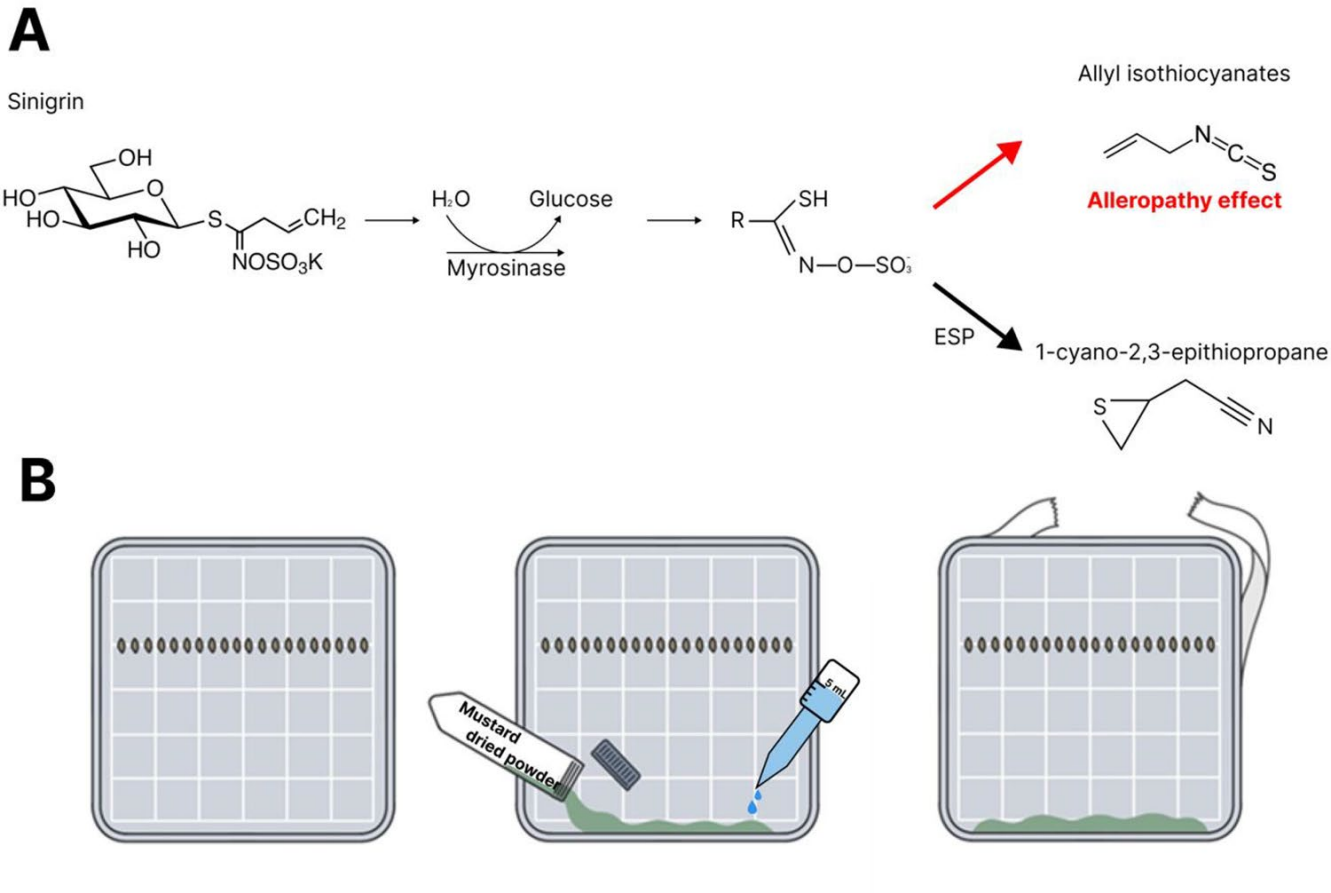
Biochemical reaction in mustard leaves revealing allelopathic effects on other species organism (**A**), schematic process of allelopathic bioassay using lettuce on square dish agar gel (**B**).

Experiments was modified as using plant leaf to plant powder on agar gel according to published method^16^. 50 mL of 2% agar gel was poured into square petri dishes (126.4 mm diameter) and cooled to solidify. The lettuce seeds (*Lactuca sativa* L.) cultivar, ‘Summer Andong’ (Kyoungshinseed, Uiseong, Korea), were used for the allelopathy test. Twenty seeds were placed horizontally on the agar surface, and the test species were then added to the agar gel (Fig. 1A). 50 mg of mustard powder and 0.5 mL of water were added to each petri dish (Fig. 1B). In case of non-treated sample, 0.5 mL of water was added. The petri dishes were sealed with parafilm to prevent gas exchanges of inside air^16^. Finally, the petri dishes were incubated at 26 °C, tilted at a 120° angle to prevent leakage of the liquid. After 24 h, the germination rates were evaluated by counting the number of germinated seeds. After three days, the shoot and root lengths were measured using ImageJ (Version 1.52; National Institutes of Health). One day after, the number of germinated individuals in each petri dish was counted and exhibited as germination rate (% of non-treatment). Subsequently, root and shoot length were measured on 3rd day after germination.

### Glucosinolate analysis

The extraction and analysis of glucosinolate in the mustard samples followed a previously described method with slight modifications of ISO 9167-1 and previous publication^21^. Briefly, 200 mg of sample powder was mixed with 2 mL of 70% methanol and 500 µL of 1 mM internal standard (glucosinalbin, isolated from *Sinapis alba* seeds) and vortexed for 10 s. The mixture was heated on a heating block at 95 °C for 10 min, then cooled on ice for 5 min. The extracts were centrifuged at 3,000 × g for 10 min. The supernatant was moved into a glass tube, and the residue was re-extracted twice with 2 mL of 70% methanol. The extracted supernatants were combined, and 1 mL was transferred to a 2 mL tube. Then, 150 µL of lead/barium acetate solution (0.5 M) was added, vortexed for 10 s, and centrifuged at 15,000 × g for 1 min. The centrifuged mixture was drained into a polyprep column filled with DEAE Sephadex A-25 (GE Healthcare, Piscataway, NJ, USA). The column was washed sequentially with 3 mL of 0.02 M pyridine acetate and 3 mL of deionized water. After the water had passed through, 500 µL of sulfatase solution (20 U mL^−1^, *Helix pomatia* Type-1, Sigma-Aldrich, St. Louis, MO, USA) was added to the column, and it was incubated overnight at room temperature. The eluent was collected in a 12 × 75 mm glass tube, vortexed for 10 s, and filtered through a 0.22 µm syringe filter into an HPLC vial. The sample was injected into an HPLC (Agilent 1100, Agilent Technologies, Palo Alto, PA, USA) equipped with a Kromail RP-C18 column (250 mm × 4.6 mm; 5 µm; 100 Å) (AkzoNobel, Bohus, Sweden) at a flow rate of 1.5 mL min^−1^. The mobile phase A (HPLC grade water) and mobile phase B (acetonitrile) were used in a gradient system: 0 min, 1.5% B; 4 min, 4% B; 20 min, 20% B; 21 min 80% B; 22 min 80% B; 24 min, 1.5% B; 26 min, 1.5% B. The UV response factors were adjusted to each glucosinolate for quantification.

### Glucosinolate hydrolysis products analysis

To extract hydrophilic glucosinolates and myrosinase from 75 mg of freeze-dried leaf mustard powder, 1.5 mL of deionized water was used. The sample was left to incubate at room temperature for 1 h, after which the mixture was centrifuged at 12,000 × g for 5 min. 500 µL of the supernatant was then transferred to a 1.5 mL polytetrafluoroethylene (PTFE) tube^22^. To perform the liquid–liquid extraction, a solvent mixture of 490 µL dichloromethane (DCM) and 5 µL phenyl isothiocyanate (dissolved in DCM at a concentration of 1 mg mL^−1^) was used. The mixture was incubated at 37 °C for 1 h, then vortexed briefly, and subsequently centrifuged at 12,000 × g for 1 min. 200 µL of the DCM layer was transferred to a vial with an insert, and 1 µL of the sample was injected into a gas chromatograph (Nexis GC-2030, Shimadzu, Kyoto, Japan) coupled to a gas chromatograph–mass spectrometer (GC/MS-QP 2020 NX, Shimadzu, Kyoto, Japan) and an autosampler with an Injector (AOC-20i PLUS, Shimadzu, Kyoto, Japan). The chromatographic separation was performed using a capillary column (DB-5MS, Agilent Technologies, Santa Clara, CA, USA; 30 m × 0.25 mm coated with 0.25 µm film). The sample was held at 40 °C for 1 min, and then the oven temperature was increased to 200 °C at 15 °C min, and further increased to 300 °C at 25 °C∙min^−1^. The injector and detector temperatures were set at 260 °C and 300 °C∙min^−1^, respectively, and the flow rate of helium carrier gas was 1.2 mL min^−1^. The AITC standard curve (0.03 µmole–0.20 µmole) was used for hydrolysis products quantification.

### Myrosinase activity and ESP nitrile formation

Nitrile formation (%) was measured to estimate the ESP activity, as ESP enhances the formation of nitriles over isothiocyanates^23^. The methods were followed previous study by Ku, et al.^24^. Nitrile formation of a leaf mustard was determined by incubating concentrated horseradish root extract with crude protein extract of the sample, and it was analyzed using GC/MS. 10 g of horseradish powder were mixed with 100 mL of 70% methanol. After vortexing the supernatant, it was centrifuged at 4000 × g for 5 min. Then, the mixture was boiled in the heating block until all the methanol solvent had evaporated. 1 mL of concentrated horseradish root extract was transferred to a 2 mL microcentrifuge tube and mixed with 25 mg of leaf mustard powder and at 12,000 × g for 3 min. The 200 µL of supernatant was transferred to a 1.5 mL PTFE tube and mixed with 1 mL of DCM. Samples were incubated at room temperature for 1 h. The mixture was then vortexed for 10 s and centrifuged at 12,000 × g for 3 min. The bottom organic layer was transferred into a vial with an inserter, and 1 µL of the sample was injected into a GC/MS. The sample was held at 40 °C for 1 min. The oven temperature was increased to 180 °C at 15 °C∙min^−1^ and then increased to 200 °C at 20 °C∙min^−1^. The injector and detector temperatures were set at 260 °C and 300 °C, respectively. The flow rate of helium carrier gas was 1.2 mL∙min^−1^ and the split ratio was 1:9. AITC standard curve (0.03 µmole–0.20 µmole) was used to quantify the hydrolysis rate of nitriles.

### Data analysis

Statistical analyses were conducted using JMP (JMP Pro software ver. 16.0, SAS Institute, Cary, NC, USA), where Tukey’s HSD test (*p* ≤ 0.05) and Pearson’s correlation analysis among the nine cultivars were performed. The correlation matrix was computed in RStudio (RStudio Inc., Boston, USA), utilizing packages such as ggplot2 and corrplot, to analyze eleven variables including sinigrin, total GSL, CETP, AITC, total GSHP, shoot length, root length, total length, germination rate, nitrile formation, and myrosinase activity. One-way ANOVA was carried out using Excel (version 2021, Microsoft Corporation, Redmond, Washington, USA). Variance components were estimated from the mean squares obtained in the analysis of variance^25^. These components, comprising error variance (VE) and genotypic variance (VG), were calculated according to a formula detailed in reference^26^. In this study, genotypic variance represented the sum of squares for the cultivars. Narrow sense heritability (*h*^2^) was estimated following the methodology described by Abu-Ellail, et al.^27^. The coefficient of variation for each trait was calculated based on the mean of the genotypes.

$${h}^{2} = VG/VE$$

### Guidelines of experimental research and field studies

Authors complied with the IUCN Policy Statement on Research Involving Species at Risk of Extinction and the Convention on the Trade in Endangered Species of Wild Fauna and Flora.

## Results

### Morphological characteristic

Studying the morphological characteristic among different cultivars is a fundamental aspect of agricultural research. Specifically, researching into the differences in harvest timing and other growth characteristics among these cultivars plays an important role in improving agricultural productivity and resource efficiency. The morphological traits of the nine leaf mustard cultivars were presented in Table 1. Two groups, early harvested cultivar and late harvested cultivar were determined according to bolting period. On the 60th day of growth, which on April 30, four cultivars including ‘Dolsan (control)’, ‘Alsami’, ‘Sundongii’, ‘Kkottolii’ were harvested. These cultivars displayed bolting rates exceeding 80% while the other five cultivar does not bolt. All four cultivars exhibited immature growth, with insufficient leaf number and leaf length development. The early harvest cultivars displayed a significantly lower average fresh weight, measuring 47.38 ± 7.09 g (*t*-test *p* < 0.05), compared to the late harvest cultivars. The reduction in fresh weight was influenced by early bolting, where reproductive development initiated before sufficient vegetative growth, resulting in a lower fresh weight.

**Table 1:** Morphological differences of early and late harvested mustard cultivars.

Cultivars	Bolting rate (%)	Leaf length (cm)	No. of leaves	Biomass (g)	Morphological images^x^	
Early harvest cultivars						
Dolsan (control)^z^	88	17 ± 2^a^	7 ± 1^b^	37 ± 12^a^		
Alsami^z^	89	17 ± 1^a^	10 ± 1^a^	49 ± 13^ab^		
Sundongii^z^	89	16 ± 0^a^	10 ± 1^a^	47 ± 9^ab^		
Kkottolii^z^	94	17 ± 0^a^	11 ± 1^a^	57 ± 13^b^		
Late harvest cultivars						
Nuttongii^y^	0	40 ± 2^a^	19 ± 1^c^	858 ± 146^ab^		
Mekomi^y^	17	38 ± 5^a^	34 ± 2^bc^	746 ± 134		
Sindongii^y^	72	38 ± 4^a^	73 ± 9^a^	747 ± 211^b^		
Zzangdolii^y^	83	33 ± 1^a^	40 ± 7^b^	968 ± 138^a^		
Ssamdolii^y^	100	40 ± 2^a^	40 ± 1^b^	904 ± 183^ab^		

^z^Four cultivars in the early-harvested group bolted within 60 days of the transplanting date.

^y^Five cultivars in the late-harvested group bolted after 60 days of the transplanting date.

^x^Horizontal bar of each image means 10 cm.

^w^Different letters indicate significant difference among cultivars contained in early or late harvested. The significances were analyzed by Tukey’s HSD test at *p* ≤ 0.05 (*n* = 12).

On the other hand, the five cultivars harvested on May 21st showed a lower bolting rate than the early-harvested cultivars, despite being harvested later. ‘Nuttongii’ did not initiate bolting, yet it was harvested due to its extended growth period exceeding the recommended 90 days, which had the potential to influence the final quality of the leaf mustard. Among the cultivar, in leaf number, ‘Sindongii’ had the highest number of 73 ± 9, and for fresh weight, ‘Zzangdolii’ had the highest at 968 ± 138.2 g. However, while other cultivars exhibit bolting after an average number of leaf mustard growth days, ‘Nuttongii’ does not show any floret formation. In this study, we harvested ‘Nuttongii’ for comparison with other cultivars.

### Allelopathy effect of volatile compounds

**Figure 2:**
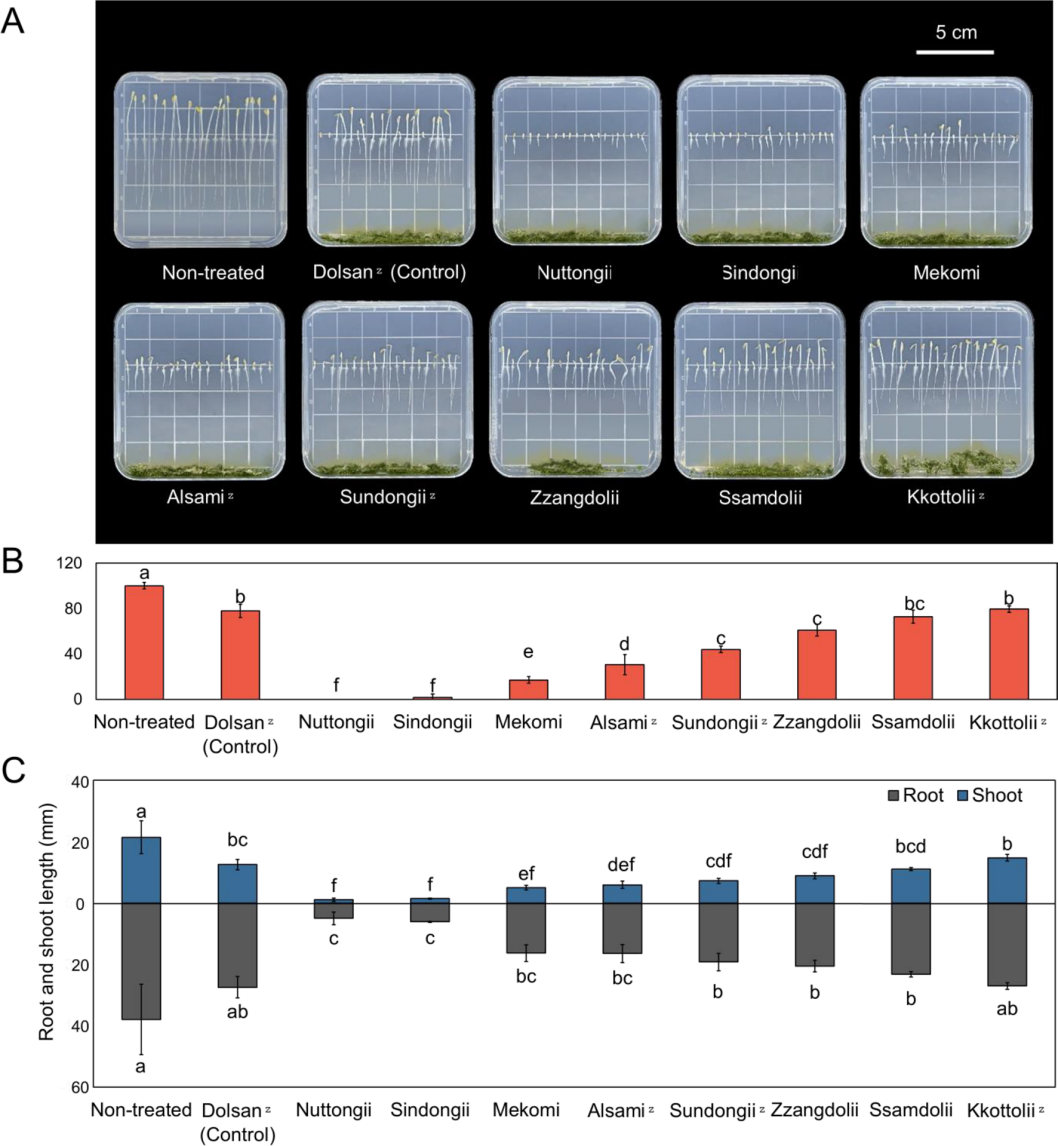
Allelopathic effects of nine mustard cultivar water extracts using freeze-dried powder treatments on lettuce (*Lactuca sativa* L.) seed germination and growth on agar gel. The represented photograph impact of leaf mustards (**A**), germination rate (% of control) (**B**) and root and shoot length (**C**). Different letters indicate significant differences among the leaf mustard treatments by Tukey’s HSD at *p* ≤ 0.05. ^Z^: Early harvested cultivars.

Bioassays assessing germination rate and initial growth of lettuce seedlings were conducted to evaluate the allelopathic potential of volatile compounds in the leaves of each mustard cultivar (Fig. 2). These volatiles significantly inhibited both germination and initial growth of lettuce seedlings, as shown in Fig. 2A, even in the absence of direct contact. The bioactive compound in mustard is known as glucosinolate, specifically sinigrin^28^. Despite the indirect contact with sinigrin, an allelopathic effect was observed in all mustard cultivar^29^. As shown in Fig. 2B, leaf mustard significantly inhibited the germination of lettuce seeds, especially with the treatments of ‘Nuttongii’, ‘Sindongii’, ‘Mekomi’, ‘Alsami’, ‘Sundongii’, and ‘Zzangdolii'. Moreover, these six cultivars also inhibit growth of lettuce seedlings (Fig. 2C). The result shows that leaf mustard powder treatments resulted in delayed seed germination and inhibited shoot and root growth, compared with non-treatment. The leaf mustard powder, initiating hydrolysis by absorbing moisture on the agar gel, released hydrolysis products into the dish. The inhibition of seed germination and growth by these hydrolysis products may be attributed to principles such as cell cycle arrest^30,31^.

### Glucosinolates and glucosinolates hydrolysis products

**Figure 3:**
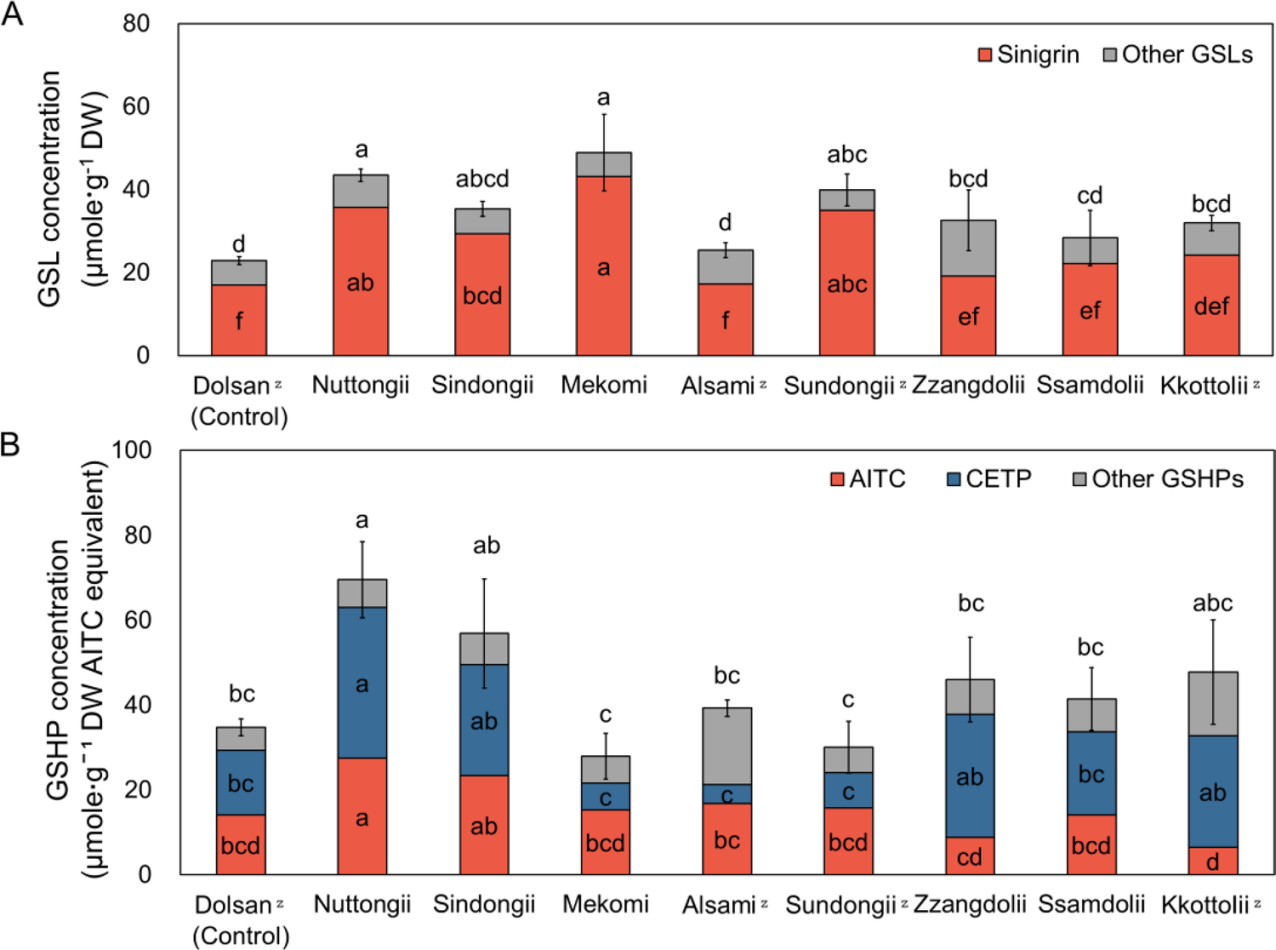
Concentration of glucosinolates (**A**) and glucosinolate hydrolysis products (**B**) in nine leaf mustard cultivars. Data were represented as mean and standard deviation for total glucosinolates or glucosinolate hydrolysis products concentration (*n* = 3). Different letters indicate significant difference of sinigrin and total glucosinolate among cultivars by Tukey’s HSD at *p* ≤ 0.05. ^Z^: Early harvested cultivars.

In this study, we analyzed the glucosinolate (GSL) concentration and their glucosinolate hydrolysis products (GSHP) in nine mustard cultivars to understand the allelopathic effect of leaf mustard, as shown in Fig. 3. Investigating both GSL and GSHP concentrations is crucial to comprehend the correlation and composition of these substances and their hydrolysis products^32^. This analysis provides insights into the compounds responsible for allelopathy. Figure 3A presents the total GSL concentration and sinigrin concentration, with error bars indicating the standard deviation of the total GSL. Sinigrin was the predominant GSL in leaf mustard, accounting for 79% of all detected GSLs. The identified GSLs include glucoiberin, glucoraphanin, sinigrin, gluconapin, 4-hydroxyglucobrassicin, glucoiberverin, glucobrassicin, 4-methoxyglucobrassicin, gluconasturin, and neoglucobrassicin. Figure 3B shows the total concentration of ten GSHPs, including AITC and CETP, with error bars for the total GSHP standard deviation. Among the GSHPs, AITC and CETP were the primary compounds, constituting 36% and 45% of the total GSHP, respectively. Detected GSHPs also include 1-cyano-2,3-epithiopropane, 1-cyano-3,4-epithiobutane, 1-cyano-4,5-epithiopentane, 3-butenyl isothiocyanate, 3-phenylpropionitrile, allyl isothiocyanate, allyl thiocyanate, iberverin, iberverin nitrile, and phenethyl isothiocyanate. ‘Mekomi’, ‘Nuttongii’, ‘Sundongii’, and ‘Sindongii’ exhibited significantly higher GSL concentrations than ‘Dolsan’ (control), with these four cultivars also showing higher sinigrin concentrations. ‘Mekomi’, ‘Nuttongii’, ‘Sundongii’, and ‘Sindongii’ showed significantly higher GSL concentration than ‘Dolsan (control)’. Those four cultivars also showed significantly higher sinigrin concentration. ‘Mekomi’ showed the highest sinigrin concentration as 43.13 ± 9.51 μmole·g^−1^ DW. GSL concentrations were highest in order of ‘Mekomi’, ‘Nuttongii’, ‘Sundongii’, ‘Sindongii’, ‘Zzangdolii’, ‘Kkottolii’, ‘Ssamdoli’, ‘Alsami’, and ‘Dolsan (Control)’. On the other hand, the order of cultivars with GSHP concentration did not correspond to the order of cultivars with GSL concentration. GSHP concentrations were highest in order of ‘Nuttongii’, ‘Sindongii’, ‘Alsami’, ‘Sundongii’, ‘Mekomi, ‘Ssamdoli’, ‘Dolsan (Control)’, ‘Zzangdolii’, and ‘Kkottolii’. ‘'Nutdongii’ exhibited the highest AITC concentration at 27.47 ± 6.46 µmole g^−1^ and CETP concentrations at 35.65 ± 10.89 µmole g^−1^ which is most significantly higher among the cultivars. ‘Mekomi’ exhibited the highest sinigrin concentration, while the content of GSHP was relatively low. This suggests that factors other than the substrate may influence the quantity of hydrolysis products.

### Myrosinase and its co-factor enzyme analysis

**Figure 4:**
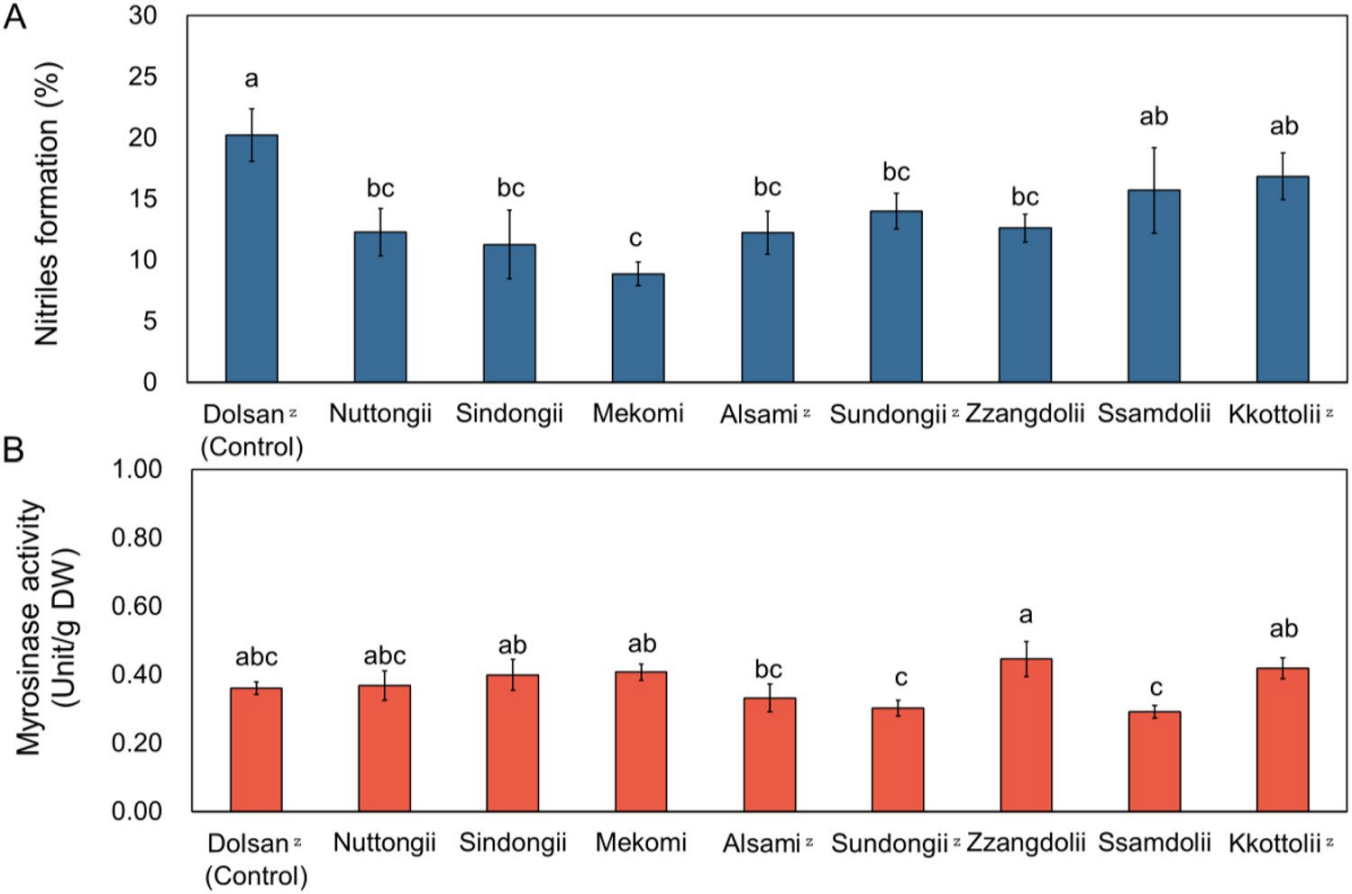
Nitriles formation (%) and myrosinase activities in nine mustard cultivars. Different letters indicate significant difference of the nitrile formation (%) and myrosinase activities among cultivars respectfully by Tukey’s HSD at *p* ≤ 0.05. ^Z^: Early harvested cultivars.

The results presented in Fig. 4 suggest that myrosinase and ESP enzyme activities significantly influence GSHP concentrations. In Fig. 4A, the nitrile formation (%) indicates the conversion rate of sinigrin into AITC or CETP, while Fig. 4B shows how myrosinase activity affects the overall concentration of GSHPs. This analysis was conducted to identify the factors contributing to the variation in GSHP concentration among cultivars that have similar levels of GSL concentrations. Each cultivar demonstrates unique myrosinase activity and nitrile formation percentage. The order of cultivars based on the highest nitrile formation (%) is as follows: ‘Dolsan (Control)’, ‘Kkottolii’, ‘Ssamdolii’, ‘Sundongii’, ‘Zzangdolii’, ‘Nuttongii’, ‘Alsami’, ‘Sindongii’, and ‘Mekomi’. Except for ‘Ssamdolii’ and ‘Kkottolii’, all other cultivars showed lower nitrile formation compared to ‘Dolsan (Control)’. Regarding myrosinase activity, the order from highest to lowest is: ‘Zzangdolii’, ‘Kkottolii’, ‘Mekomi’, ‘Sindongii’, ‘Nuttongii’, ‘Dolsan (Control)’, ‘Alsami’, ‘Sundongii’, and ‘Ssamdolii’.

### Correlation between leaf mustard and their allelopathy effect

**Figure 5:**
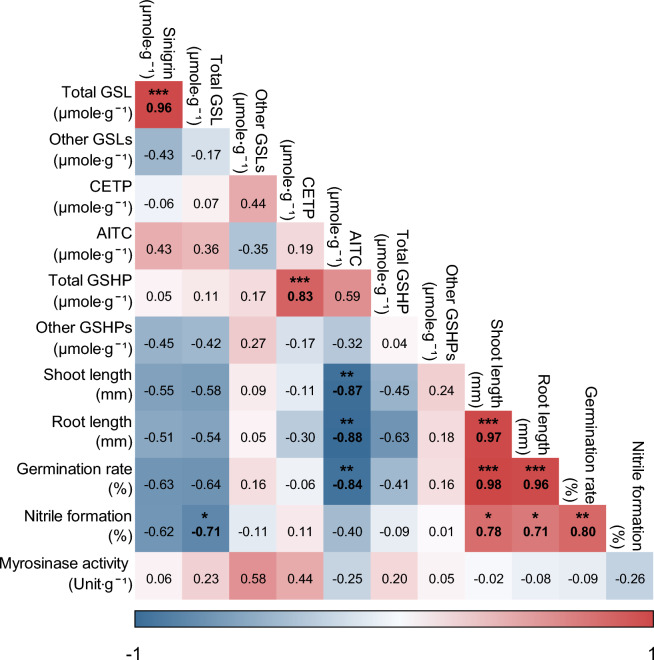
Pearson correlation matrix amomg the leaf mustard compounds and their allelopathic impact on lettuce (*Lactuca sativa* L.) growth parameters. Colors represent different correlation strengths, with blue and red representing strong negative and positive correlations, respectively (*n* = 9). *, ** and *** indicated 5%, 1% and 0.1% levels of significance, respectively.

Pearson correlation analysis was conducted to examine the relationship between the allelopathic effects, and the compounds analyzed in leaf mustard. It is known that the Brassicaceae family contains allelochemicals in ITC (isothiocyanate) form, which can inhibit weed growth^33^. The study examined the impact of different compounds (GSL, other GSL, CETP, AITC, total GSHP, and other GSHP) on lettuce seed parameters (shoot length, root length, germination rate) and visualized their interactions through a heat map (Fig. 5).

The analysis revealed a strong positive correlation between 'sinigrin' and 'total GSL' (*r* = 0.964, *p* < 0.001) and, between 'CETP' and 'total GSHP' (*r* = 0.830, *p* < 0.01), indicating these are major contributors to GSL and GSHP, respectively, across all cultivars. However, 'sinigrin', 'total GSL', 'CETP', and 'total GSHP' did not show allelopathic effects on lettuce seeds. The study also confirmed that 'AITC,' derived from sinigrin, significantly inhibits lettuce seed growth even at lower concentrations than CETP. AITC concentrations were found to be significantly negatively correlated with 'shoot length' (*r* = − 0.87, *p* < 0.01), 'root length' (*r* = − 0.88, *p* < 0.01), and 'germination rate' (*r* = − 0.84, *p* < 0.01) of lettuce. Additionally, a positive correlation between 'nitrile formation' and 'shoot length' (*r* = 0.78, *p* < 0.05), 'root length' (*r* = 0.71, *p* < 0.05), and 'germination rate' (*r* = 0.08, *p* < 0.01) indicates that higher nitrile formation leads to a lower rate of ITC generation with allelopathic activity from the substrate GSL.

### Determination of heritability of allelopathic effect and allelochemicals

The impact of cultivars, which is genetic factors, on lettuce seed allelopathy has not been investigated^18^. To understand the genetic influence on allelopathic effect and compounds of leaf mustard, we conducted an analysis of variance (ANOVA) to calculate and quantify each factor and trait. Table 2 revealed highly significant differences in all 11 traits analyzed through ANOVA at the *p* < 0.001 level, with *h*^2^ exceeding 0.7. This research suggests that allelopathic effects are influenced by genetic factors. Furthermore, the composition of each cultivar was also found to be determined by genetic factors. These results indicate that the cultivar is a key determinant of both compounds and allelopathic effects.

**Table 2:** One way ANOVA for the studied traits of nine leaf mustard cultivar.

Compound	*h*^2^	F value
Allelopathy effect on lettuce seeds		
Germination rate	0.99***	133.39
Shoot length	0.97***	73.62
Root length	0.94***	38.08
Compounds of leaf mustard		
Sinigrin	0.88***	16.35
AITC	0.84***	11.45
CETP	0.84***	11.7
Total GSL	0.81***	9.35
Total GSHP	0.77***	7.74
Myrosinase activity	0.76***	7.11
Nitrile formation	0.76***	7.32

***indicates significantly lower than *p* < 0.001.

*h*^2^: Narrow sense heritability.

## Discussion

### Potential allelopathic mustard cultivar based on AITC: implications for future cultivation and breeding

AITC a biofumigant recognized in the United States^34^, is a key component released by leaf mustard, making it a valuable resource in sustainable weed management practices^35^. The adoption of allelopathic plants, like leaf mustard, represents a significant step toward environmentally friendly and sustainable weed control^36^. Utilizing the researched leaf mustard water potential values^37^, we calculated the concentration of AITC per 100 g of leaf mustard fresh weight in this study.

In the late-harvested group, ‘Nuttongii’ had the highest AITC concentration per 100 g fresh weight (246.16 ± 46.79 µmole 100 g^−1^), surpassing ‘Zzangdolii’, which had the highest fresh weight but the lowest AITC content at 83.29 ± 2.92 µmole 100 g^−1^ (FW). Remarkably, ‘Nuttongii’ not only exhibited elevated AITC levels but also significantly inhibited germination rates and root and shoot lengths, corroborating previous studies on AITC's allelopathic effects and its correlation with AITC concentrations^38^. This suggests that ‘Nuttongii’ holds potential as an effective herbicide based on its AITC concentration. Previous study found that the mechanism of allelopathy in horseradish involves the release of allyl isothiocyanate compounds from its root extract, which, at increasing concentrations, inhibit onion root growth, disrupt the cell cycle, and increase cell death, thereby demonstrating a concentration-dependent inhibitory effect^39^. Additionally, ‘Nuttongii’ may achieve higher fresh weight if left unharvested due to late bolting. Harvesting practices for ‘Nuttongii’ differed from other cultivars, potentially leading to an underestimated biomass. Future studies should consider cultivating ‘Nuttongii’ until bolting for more accurate sinigrin measurements. Our research revealed that the precursor of sinigrin had minimal impact on allelopathic activity, contrary to previous studies suggesting its influence through indirect contact. Previous research has been unable to deduce a precise cause-and-effect relationship through statistical correlation analysis^18^. Given these results, Consequently, in the future, cultivation and breeding efforts should focus on AITC, given its physiological significance as a bioactive compound.

### Enhanced allelopathic effect in leaf mustard: significance of nitrile formation and ESP activity

A critical aspect of this study involves exploring the genetic determinants of AITC production and nitrile formation. Allelochemicals like AITC are produced when sinigrin undergoes hydrolysis in the presence of water, facilitated by the myrosinase enzyme. This process transforms sinigrin into hydrolysis products including AITC^40^. However, previous research has largely overlooked the crucial role of nitrile formation in AITC production and the CEPT mechanism. Our findings underscore the substantial impact of these factors on allelopathic effects, revealing a robust genetic underpinning, as highlighted in Table 2.

**Figure 6:**
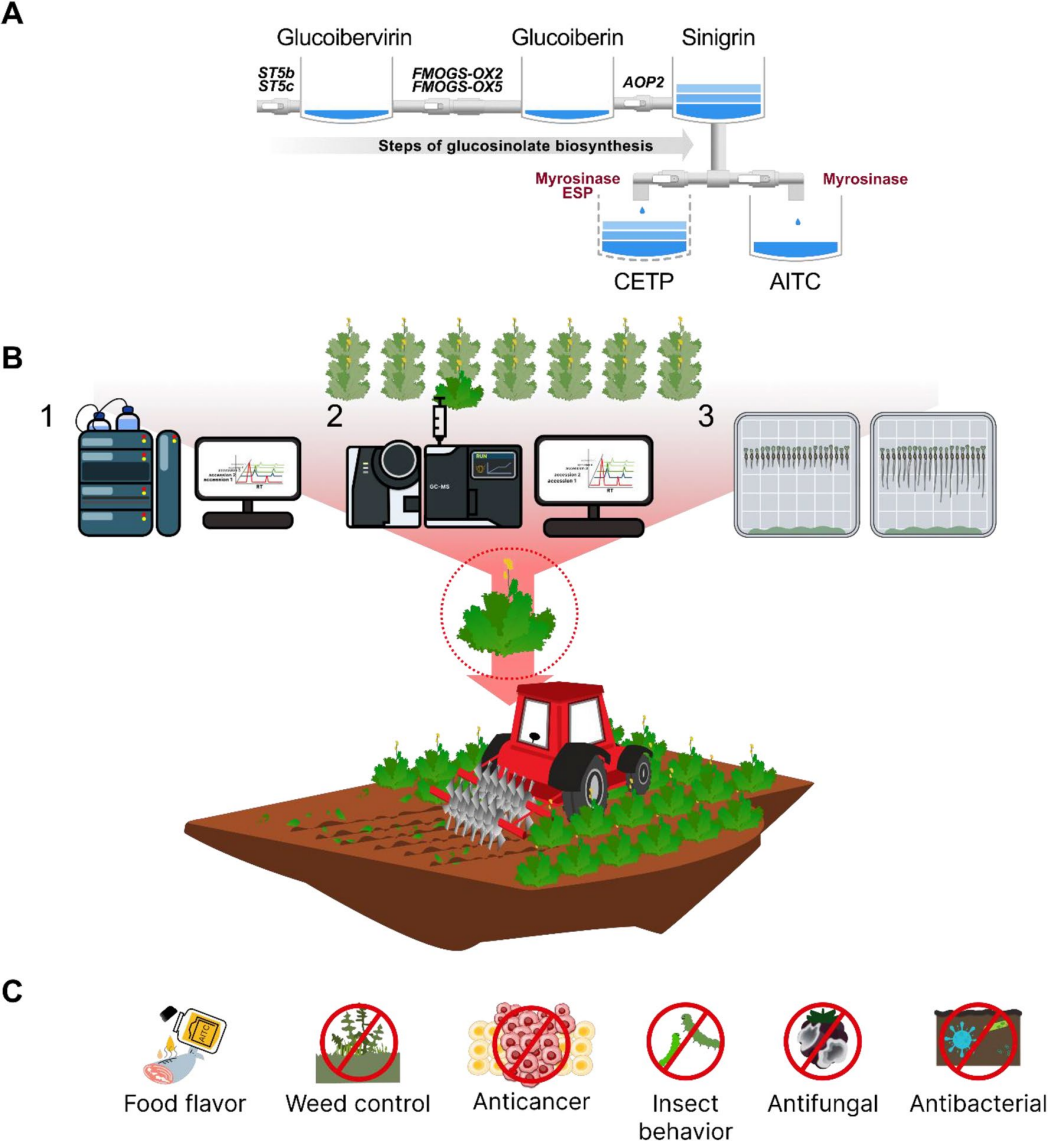
Glucosinolate biosynthesis and the formation of glucosinolate hydrolysis products influenced by the presence or absence of the epithiospecifier protein (ESP) of leaf mustard (**A**), selection of the optimal leaf mustard cultivar based on bioactive compound analysis, integrated into agricultural practices for effective biofumigation management (**B**), and potential application of enhanced allyl isothiocyanate (AITC) containing leaf mustard (**C**).

While earlier mustard cultivation research primarily focused on sinigrin content^41^, our investigation into sinigrin and its conversion products reveals a surprising lack of correlation. Our study has revealed a correlation between AITC levels and the enhancement of allopathic effects, indicating that varieties genetically regulated by the *ESP* gene may possess higher allelopathic capabilities. (Fig. 6A). Consequently, breeding for high AITC content should prioritize reducing ESP activity^7^. Our findings also demonstrate the significant contribution of AITC to the physiological activity observed in mustard plants. Utilizing advanced AITC measurement techniques and nitrile formation screening assays^18,24,42^, we have identified promising candidates mustard leaf ‘Nuttongii’ for enhancing sustainable agricultural production. By manipulating genes related to AITC synthesis and nitrile formation, we can expedite the development of cultivars with enhanced allelopathic properties. Our research suggests that CRISPR-Cas9 is an efficient tool for enhancing desired glucosinolates, including targeting the ESP gene to increase bioactive allelochemicals from sinigrin. This method not only enhances desired traits but also minimizes unintended genetic changes, ensuring the safety and predictability of the breeding process.

In the face of sustainability challenges, eco-conscious agricultural practices are increasingly important Cultivars produce higher AITC levels with reduced nitrile formation, as our research suggests, can significantly lessen the dependence on chemical herbicides (Fig. 6B). This shift can improve soil health, decrease chemical runoff, and reduce harm to non-target organisms, contributing to more environmentally friendly farming methods.

### Biofumigation agricultural practices and management strategies

Incorporating brassica species as cover crops within agricultural rotations presents a dual-functional strategy for soil conservation and sustainable pest and weed management^43^. This practice, termed biofumigation, necessitates strategic planning and precise execution for optimal efficacy. Central to this strategy is the selection of appropriate biofumigant crops, such as mustard, which are known for their ability to release volatile compounds with pesticidal properties. A meticulously designed crop rotation scheme is imperative for diversifying the production cycle, thereby mitigating the risk of pest and pathogen build-up and enhancing the biofumigation effect. The effective use of cover crops for biofumigation, particularly during their decomposition phase, requires meticulous planning regarding the timing of their incorporation into the soil and an understanding of existing soil conditions. This careful approach is crucial for maximizing their biofumigation potential. Notably, the concentration of glucosinolates, key compounds in biofumigation, is affected by variables such as the growing season^44,45^ and potential insect damage, as indicated in studies^46–51^. These factors underscore the importance of strategic crop management to enhance biofumigation efficacy for ecofriendly cultivation.

### Beyond fumigation: unveiling the multifaceted potential of AITC in food and medicine

AITC treatment has been reported to be effective against various pathogens and weeds under warm conditions in sandy soil when applied before planting crops^52,53^. It possesses notable fumigant properties effective against a range of soil-borne pathogens and weeds^54^, as demonstrated in Fig. 6C. The United States Food and Drug Administration has designated AITC as a 'Generally Recognized as Safe' (GRAS) substance, underscoring its potential as a benign food additive. This compound's diverse physiological properties open up a multitude of applications in agricultural practices.

One of the primary advantages of AITC is its function as a natural herbicide, offering an environmentally sustainable alternative to traditional synthetic herbicides. This aspect of AITC is elaborated further in the preceding sections of this paper. Additionally, AITC has been observed to possess significant antipathogenic properties, particularly in the context of postharvest plant disease management. Studies have indicated its efficacy in combatting fungal infections, notably reducing decay in perishable fruits such as strawberries, blackberries, blueberries, raspberries, and other similar crops^38,55,56^.

In the field of food engineering, the utilization of AITC emerges as a promising strategy for augmenting both food preservation and flavor enhancement. This approach is in line with the current trend towards developing food products that are both safer and more flavorful. A key element in harnessing AITC's potential involves the targeted manipulation of the ESP gene, a critical factor in the biosynthesis of AITC from its precursor, sinigrin. Advances in genetic engineering techniques enable the complete conversion of sinigrin into AITC, which is notable for its potent aroma, estimated to be around 300 times stronger than that produced by CETP^57^. This technology is crucial for foods like sauces and condiments that need distinct aromas. It enables healthier, naturally flavored products, reducing artificial additives and aligning with consumer trends towards natural ingredients. Additionally, AITC shows promise as an antibacterial agent, potentially improving food safety. This is especially relevant in food safety research, where the prevention of pathogen outbreaks in salad vegetables is a significant concern^58^. AITC's application could enhance the safety of these vegetables, contributing to safer food consumption.

In the medical field, AITC has garnered attention due to its promising medicinal properties, particularly its potential as an anticancer agent. highlighted by its ability to induce apoptosis, inhibit carcinogenesis, enhancing detoxifying phase II enzymes^24^ and exert anti-inflammatory effects, make it a compound of considerable interest in the medical community^59^. The AITC-enhanced mustard, with its potential as a therapeutic agent in cancer treatment, can be linked to further research, particularly through clinical studies.

Additionally, in the field of ecology, AITC may play a crucial role in deepening our understanding of its ecological impact, particularly concerning plant–insect interactions and ecosystem dynamics. This compound, naturally present in cruciferous plants, serves as a bioactive substance with potential influences on various ecological processes. AITC's role in plant–insect interactions is significant. It functions as a natural pest deterrent, offering an ecological advantage to plants that produce it. This characteristic can alter the and feeding preferences and growth of insect pests^60^. This is evident in the modulation of feeding behaviors and growth patterns in insect pests, as indicated in previous studies examining the effects of glucosinolates, a class of compounds to which AITC belongs, on insect feeding^56^.

In conclusion, our research provides a clear target for precision breeding techniques, leveraging genetic factors influencing AITC production and nitrile formation, ultimately advancing eco-conscious agriculture and promoting sustainability in farming practices, while opening doors to cross-disciplinary applications of AITC modulation.

## Conclusion

In conclusion, 'Nuttongii' cultivars exhibit notably elevated levels of AITC and pronounced allelopathic effects compared to other varieties. Our research underscores the superior allelopathic properties of selected 'Nuttongii' plant varieties, which can be attributed to genetic diversity influencing enzyme activities. In summary, this approach promotes environmentally friendly agriculture and sustainable farming practices, while also facilitating interdisciplinary applications in AITC modulation.

## Data Availability

Data is provided within the manuscript or supplementary information files.
